# Correlation Between Cardiac Troponin Serum Concentration and Selected Parameters of Subclinical Cardiovascular Dysfunction in Patients With and Without Arterial Hypertension: Retrospective Cross-Sectional Analysis of Real-World Data

**DOI:** 10.3390/jcm14175961

**Published:** 2025-08-23

**Authors:** Grzegorz K. Jakubiak, Monika Starzak, Natalia Pawlas, Artur Chwalba, Agata Stanek, Grzegorz Cieślar

**Affiliations:** 1 Department of Pharmacology, Faculty of Medical Sciences in Zabrze, Medical University of Silesia, Jordana 38 St., 41-808 Zabrze, Poland; n-pawlas@wp.pl (N.P.); artur.adam.chwalba@gmail.com (A.C.); 2 Department of Internal Medicine, Angiology and Physical Medicine, Faculty of Medical Sciences in Zabrze, Medical University of Silesia, Batorego 15 St., 41-902 Bytom, Poland; ipl.m.starzak@gmail.com (M.S.); cieslar1@tlen.pl (G.C.); 3 Department of Internal Medicine, Metabolic Diseases and Angiology, Faculty of Health Sciences in Katowice, Medical University of Silesia, Ziolowa 45/47 St., 40-635 Katowice, Poland; astanek@tlen.pl

**Keywords:** cardiac troponin, hypertension, atherosclerosis, pulse wave velocity, intima–media thickness, ankle–brachial index, toe–brachial index, echocardiography

## Abstract

**Background**: High-sensitivity cardiac troponin T serum concentration (hs-cTnT) measurement is a well-established tool in the diagnosis of acute cardiovascular (CV) disease. It remains unclear whether resting hs-cTnT could be useful for screening the status of the CV system. The purpose of this study was to compare the correlation between hs-cTnT, determined in patients without clinical symptoms of acute illness, and selected parameters of subclinical CV dysfunction in relation to the coexistence of arterial hypertension (AH). **Methods**: In total, 101 patients were included in the analysis. The following methods were used to assess the CV system: transthoracic echocardiography, Doppler ultrasonography of the carotid and lower extremity arteries with intima–media thickness (IMT) measurement, pulse wave velocity (PWV), central blood pressure measurement, ankle–brachial index (ABI), and toe–brachial index (TBI). **Results**: In patients with AH, significant correlations were found between hs-cTnT and maximal velocity of tricuspid regurgitation (*R* = 0.397; *p* = 0.003), left atrium volume index (LAVI) (*R* = 0.39; *p* = 0.002), and IMT in carotid arteries (cIMT) (*R* = 0.4; *p* = 0.001), common femoral arteries (cfIMT) (*R* = 0.384; *p* = 0.004), and superficial femoral arteries (sfIMT) (*R* = 0.352; *p* = 0.01), as well as PWV (*R* = 0.63; *p* < 0.001), central systolic blood pressure (cSBP) (*R* = 0.34; *p* = 0.006), central pulse pressure (cPP) (*R* = 0.354; *p* = 0.004), and ankle–brachial index (ABI) (*R* = −0.28; *p* = 0.024). In multivariate analysis, the relationship between subclinical CV dysfunction and hs-cTnT remained significant for LAVI, cSBP, cPP, and ABI, as well as showing borderline significance for sfIMT. In patients without AH, only the relationship between hs-cTnT and ABI was significant. According to interaction analysis, AH significantly influenced the relationship between hs-cTnT and cSBP, cPP, and sfIMT. **Conclusions**: Resting hs-cTnT correlates significantly with selected parameters of subclinical CV dysfunction in patients with AH. This relationship is clearly weaker in patients without AH. Further research is needed, especially prospective studies on a larger group of patients.

## 1. Introduction

There is no doubt that cardiac troponin serum concentration (cTn) is a basic tool in laboratory diagnostics in patients with suspected acute cardiovascular (CV) disease. The determination of cTn plays a key role in the diagnosis of acute coronary syndrome [1]. In patients with pulmonary embolism, cTn testing is crucial in the risk stratification for hemodynamic instability [2]. cTn also increases in other diseases, such as heart failure [3], tachyarrhythmias [4], or acute aortic syndromes [5], or serious non-cardiac diseases, such as sepsis [6] or respiratory failure [7].

However, the question is increasingly being asked whether resting cTn, determined without the clinical context of suspected acute CV disease, could be useful for screening the status of the CV system and, if so, how such tests should be conducted and how their results should be interpreted [8].

Increased blood pressure and associated left ventricular overload may contribute to the occurrence of biochemical signs of myocardial damage [9]. According to the recommendations of the European Society of Hypertension, tools dedicated to the assessment of subclinical CV dysfunction in the course of arterial hypertension (AH) include measurement of pulse wave velocity (PWV), assessment of intima–media thickness (IMT), and measurement of the ankle–brachial index (ABI) or the left ventricle mass index (LVMI) [10]. A valuable supplement to the ABI measurement is the toe–brachial index (TBI) [11]. It is worth noting that although the classic site of IMT measurement is the common carotid artery (cIMT), attention has also been paid to the value of IMT measurement in other vascular beds, such as the common femoral artery (cfIMT) and the superficial femoral artery (sfIMT) [12].

The purpose of this study was to compare the correlation between resting high-sensitivity cardiac troponin T serum concentration (hs-cTnT) levels determined in patients without symptoms of acute illness and selected parameters of subclinical CV dysfunction in patients with and without AH. The preliminary results of the data analysis on which this publication is based were presented at the 34th European Meeting on Hypertension and Cardiovascular Protection (European Society of Hypertension, Milan, Italy, 23–26 May 2025) [13,14].

## 2. Materials and Methods

### 2.1. Study Population

The study population included patients hospitalized in the Department of Internal Medicine, Angiology and Physical Medicine of the Medical University of Silesia in Katowice (Poland) in the period between June 2022 and February 2024 who had undergone non-invasive CV assessment and hs-cTnT determination. Patients with any acute illness or exacerbation of chronic disease within the month preceding their admission to the hospital, tachyarrhythmia, features of decompensated heart failure, respiratory failure, water/mineral balance disturbances, anemia, infection, diagnosed neoplasm, autoimmune disease, or chronic inflammatory disease were excluded.

Patients included in the analysis were classified into the group with or without AH, based on interview data and medical history, taking into account currently used medications, as well as on the basis of regular blood pressure measurements during hospitalization, which is standard in an internal medicine ward.

### 2.2. Diagnostic Procedures

An arterial Doppler ultrasound was obtained using an RS80 EVO device (Samsung Medison Co., Ltd., Gangwon-do, Republic of Korea) with a linear probe LA4-18B (ultrasound of carotid arteries) or a linear probe LA3-12A (ultrasound of lower extremity arteries). Doppler ultrasonography of the carotid arteries and lower limb arteries was performed in accordance with commonly available recommendations [15,16,17]. IMT was measured manually within the common carotid artery one centimeter below the bifurcation, within the common femoral artery one centimeter above the bifurcation, and within the superficial femoral artery one centimeter below the bifurcation. Each measurement was performed three times, and a mean value was documented in the medical history. For each location, the arithmetic mean of the values obtained for the right and left side was considered for the final analysis.

Transthoracic echocardiography was performed with a Vivid E9 device (GE HealthCare Ultrasound, Chicago, IL, USA) using an M5S-D transducer. The measurements were made in standard projections in accordance with applicable recommendations [18].

The ABI was measured bilaterally using a Dopplex^®^ Ability Automatic ABI System (Huntleigh, Healthcare Ltd., Cardiff, UK). The TBI was measured bilaterally using a Dopplex DMX Digital Doppler (Huntleigh, Healthcare, Ltd., Cardiff, UK). The arithmetic mean of the ABI values measured on each side and the arithmetic mean of the TBI values measured on each side were taken as the ABI and TBI values considered for final analysis.

Central blood pressure parameters, such as central systolic blood pressure (cSBP), central diastolic blood pressure (cDBP), and central pulse pressure (cPP), and PWV were measured with Sphygmocor XCEL^®^ (AtCorMedical, Sydney, Australia).

All procedures were performed with the patient in thermal comfort conditions, at least 10 min after remaining at rest in the supine position.

All laboratory tests were performed in the Laboratory of the Specialist Hospital No. 2 in Bytom, in which the Department of Internal Medicine, Angiology and Physical Medicine is located. hs-cTnT was determined with electrochemiluminescence using the Elecsys Troponin T hs STAT kit (Roche Diagnostics GmbH, Mannheim, Germany). The minimum detectable concentration of hs-cTnT is 3 pg/mL. According to the kit manufacturer, the upper reference limit (URL) (99th percentile) for troponin T is 14.0 pg/mL and the 95% confidence interval is 12.7–24.9 pg/mL.

### 2.3. Statistical Analysis

Qualitative variables were presented as the number of variants and percentages. For quantitative variables, the Shapiro–Wilk test or Kolmogorov–Smirnov test was used along with visual analysis of histograms to assess whether there was evidence that the distribution of a given variable differed significantly from the normal distribution. For variables whose distribution significantly differed from the normal distribution, the median was used as a measure of central tendency and the first quartile (Q1) and third quartile (Q3) were used as a measure of dispersion. For other variables, the mean and standard deviation (SD) were used. To test the significance of differences between groups in quantitative variables, the Mann–Whitney U test was used for variables whose distribution differed significantly from the normal distribution, or the Student’s *t* test for variables whose distribution did not differ significantly from the normal distribution. In the case of qualitative variables, the χ^2^ test was used to compare the significance of differences between subgroups. Spearman’s rank correlation test was used to examine correlations. A value of *p* < 0.05 was considered statistically significant.

To better understand the observed relationships, taking into account confounding factors, we used the generalized linear model. The model was adjusted for age, sex, body mass index (BMI), waist-to-height ratio (WHtR), total cholesterol serum concentration (TC), low-density lipoprotein cholesterol serum concentration (LDL-C), high-density lipoprotein cholesterol serum concentration (HDL-C), triglyceride serum concentration (TG), non-HDL-C, and smoking, which were chosen based upon correlation analyses as the significant variables. The ABI and maximal velocity of tricuspid regurgitation (TRV_max_) were log-transformed due to the non-normal distribution. The multivariable adjusted model was assessed including the interaction term between AH and hs-cTnT value.

## 3. Results

### 3.1. General Characteristics of the Study Population

The final analysis included 101 patients. It is worth noting that the majority of the study population were women (62.38%). More than half of the study participants were current (22.77%) or former smokers (32.67%). In the case of three people (2.97%), there were no data in the medical records regarding attitudes towards tobacco smoking. The majority of the study population were people with diagnosed AH (64.36%). Diabetes or prediabetes was diagnosed in a significantly smaller percentage of the study population (27.72% and 14.85%, respectively). A relatively small percentage of the study population had previously been diagnosed with atrial fibrillation (9.9%) or chronic kidney disease (3.96%). It should be emphasized that none of these individuals were undergoing renal replacement therapy, and none of the patients had an estimated glomerular filtration rate (eGFR) value lower than 45 mL/min/1.73 m^2^. A high percentage of the study population was diagnosed with atherosclerosis (66.34%). It is worth emphasizing that the diagnosis of atherosclerosis should be understood as evidence of the presence of atherosclerotic plaque according to imaging tests in any vascular bed, whereas the vast majority of these were hemodynamically insignificant lesions in asymptomatic patients. Only a small percentage of the study population had been diagnosed with atherosclerotic cardiovascular disease (ASCVD). In particular, it should be noted that only a few individuals had a history of myocardial revascularization (5.94%) or stroke (2.97%). Baseline characteristics of the study population are presented in Table 1.

### 3.2. Results of the Cardiovascular System Assessment in the Study Population

Table 2 presents the measurement results of selected parameters relating to the condition of the CV system. It should be noted that although the study population included people with abnormal test results relating to the assessment of the CV system, the vast majority of the values of the assessed parameters were within the range considered normal.

### 3.3. Differences Between Individuals With and Without Arterial Hypertension

Table 3 presents data on basic characteristics and selected biochemical test results in the group of patients with and without AH. People with AH were characterized by significantly higher BMI, WHR, and WHtR values. Among people with AH, a significantly lower share of women was also found than in the group of people without AH. Apart from significantly higher TG levels in patients with AH, there were no significant differences in lipid profile parameters between the groups. A significantly higher percentage of people using statins in the group with AH compared to those without AH should be taken into account. Among people with AH, significantly higher uric acid levels and percentage of glycated hemoglobin were found than in normotensive subjects.

Table 4 presents the parameters related to the assessment of the CV system status in patients with and without AH. For most parameters, more severe features of subclinical CV dysfunction were found among patients with AH. Only in the cases of TRV_max_, cDBP, and TBI was there no significant difference between people with and without AH.

It should be noted that TRV_max_ values were missing for 23 individuals (22.77%) (12 patients with AH and 11 without AH). This was because these individuals did not have tricuspid valve regurgitation detectable on echocardiography, which would have allowed for reliable measurement. However, each patient was assessed in this respect. In the case of TRV_max_, the absence of a value is therefore not true missing data, but information showing that TRV_max_ was unmeasurable. The percentage of individuals with missing cIMT, cfIMT, and sfIMT values was lower (3.96%, 11.88%, and 12.87%, respectively). Among these individuals, Doppler ultrasound was not performed during hospitalization. PWV was not measured in three subjects (2.97%). The LAVI and LVMI values were missing for one person due to the lack of anthropometric measurements in the medical records that would allow for the calculation of body surface area.

### 3.4. Correlation Between Subclinical Cardiovascular Dysfunction in Individuals With and Without Arterial Hypertension

Among people with AH, hs-cTnT was shown to be significantly correlated with LAVI, TRV_max_, cIMT, cfIMT, sfIMT, cSBP, cPP, PWV, and ABI. Among people without AH, hs-cTnT was shown to correlate significantly only with IMT values in the femoral arteries, but interestingly, the correlation in this case was even stronger than in people with AH. The complete results for the correlation between hs-cTnT and selected parameters of subclinical CV dysfunction in people with and without AH are presented in Table 5.

### 3.5. Multivariate Analysis

To examine differences in the correlation between hs-cTnT concentrations and parameters related to CV dysfunction between patients with and without AH, an interaction analysis test was constructed for the entire cohort. A significant interaction was found for cSBP and cPP, as well as sfIMT with borderline significance. The test results are presented in Table 6.

In the subgroup of individuals without AH, only the relationship between ABI and hs-cTnT was significant. The full list of model parameters obtained for the subgroup of people without AH is presented in Table 7.

In the subgroup of individuals with AH, the relationship between subclinical CV dysfunction and hs-cTnT remained significant for LAVI, cSBP, cPP, and ABI, as well as for sfIMT with borderline significance. The full list of model parameters obtained for the subgroup of people without AH is presented in Table 8.

## 4. Discussion

### 4.1. Differences Between Patients With and Without Arterial Hypertension

When interpreting our results, it should be remembered that the group of patients with AH studied by us consisted primarily of people who did not require modification of antihypertensive treatment during hospitalization at the Department of Internal Medicine, Angiology and Physical Medicine, and only a small percentage of patients had concomitant ASCVD or chronic kidney disease (none had end-stage renal failure). Despite this, in the case of almost all parameters related to the severity of subclinical CV dysfunction, a significantly higher severity of these features was found in patients with AH. These results are fully consistent with expectations, as most of the parameters used in this study are recognized tools for assessing subclinical organ dysfunction in the course of AH.

In terms of echocardiographic assessment, people with AH were characterized by significantly higher LVMI values, which is associated with myocardial remodeling in the course of AH [19]. The E/E’ and LAVI values were significantly higher among people with AH, which is associated with a tendency to develop left ventricle diastolic dysfunction [20]. On the other hand, no significant difference was found in TRV_max_, which is a marker of diastolic dysfunction, and its increased value indicates the probability of pulmonary hypertension [21]. Perhaps the lack of difference in this parameter results from the low level of subclinical CV dysfunction in the course of AH, because the LVMI value was only above the norm in a few people. In the study conducted by Yerlikaya-Schatten et al., TRV_max_ was only slightly increased [2.9 (2.5–3.3) m/s] in the population of women with preeclampsia, but a clear left ventricular hypertrophy was found in the studied population [septal thickness 12 (10–13) mm] [22]. In the studied population, the LV EF value was statistically significantly lower among people with AH. It should be emphasized, however, that although statistical significance was achieved, from a clinical point of view, the difference in the median value in both groups was small (55% vs. 58%). The worsening of systolic function also contributes to the pathophysiology of AH [23].

Arterial stiffness assessed with PWV and cPP were significantly higher in the subgroup of hypertensive patients than in those without AH. This is consistent with the results of other researchers [24,25].

It is worth noting that the IMT value is significantly higher in people with AH than in people without AH, which is associated with increased subclinical atherosclerosis in individuals with AH. It is worth noting that it applies to the measurement made not only in the carotid artery but also in the femoral arteries. The importance of IMT measurement in the femoral arteries was already emphasized by us in one of our previous publication [12].

The observed differences between patients with and without AH could also have been caused by the significantly worse results of the subgroup with AH in terms of the coexistence of additional CV risk factors, such as diabetes or obesity. This coexistence is not surprising, because arterial AH, hyperglycemia, obesity, and atherogenic dyslipidemia constitute the clinical picture of the metabolic syndrome [26]. On the other hand, it should be remembered that the purpose of this study was not to compare people with and without AH, but to compare the correlation between hs-cTnT and features of subclinical CV dysfunction in these two patient populations.

### 4.2. Correlations Between Cardiac Troponin Serum Concentration and Features of Subclinical Cardiovascular Dysfunction in Patients With and Without Arterial Hypertension

To the best of our knowledge, no studies have been conducted to date to examine the correlation between resting cTn, determined without the clinical context of acute illness, and various parameters related to subclinical CV dysfunction in patients with and without AH. However, there is growing interest in the potential use of resting cTn as a screening marker of CV status and CV risk.

Alanis et al. recently reported the results of a large study that showed that increased arterial stiffness and subclinical atherosclerosis were associated with increased resting cardiac troponin I levels using high-sensitivity assays [27]. This is consistent with our findings, although our results are limited to hypertensive individuals and we used troponin T. On the other hand, according to other results presented by the same research team, increased vascular aging predisposes patients to the diagnosis of AH [28].

The issue of the relationship between resting cTn and arterial stiffness has also been studied in other patient populations. According to Blachut et al., resting cTn correlates significantly with PWV value in patients with systemic lupus erythematosus [29]. Similar conclusions were obtained by Sabio et al., whose study included women diagnosed with systemic lupus erythematosus and with normal renal function [30]. Our results confirm a quite strong correlation between hs-cTnT and PWV in patients with AH, but they indicate that such a relationship is primarily the result of confounding factors, as the relationship did not remain significant in the multivariate analysis model. Studies conducted so far have shown that both cTn and PWV are clearly correlated with age [31,32].

Vigen et al. presented the results of a large study that found that increasing resting cTn correlated with greater severity of CV dysfunction, including echocardiographic parameters. However, differences between troponin T and troponin I were observed. It should be noted, however, that their study was conducted in the general population, not in individuals with established AH [33].

An interesting conclusion worthy of additional comment is that among people without AH, in multivariate analysis only the relationship between hs-cTnT and ABI value proved to be significant. It is worth discussing that among individuals without AH, regression analysis revealed a significant relationship between hs-cTnT and ABI, although no significant correlation was found between these parameters. This suggests a nonlinear relationship between the variables studied. It is worth noting that, according to current knowledge, the relationship between ABI and CV risk is U-shaped, because both reduced and increased ABI values are associated with increased CV risk [34]. ABI was shown to be a valuable indicator of CV risk. According to the results presented by Myslinski et al., only ABI is a better predictor of the occurrence of a first-ever acute coronary syndrome than LVMI, IMT, lipid profile parameters, eGFR, or high-sensitive C-reactive protein. It should be emphasized that the aforementioned study included only individuals with AH [35]. According to another study, ABI is independently associated with an increased risk of sudden cardiac death in a middle-aged general population [36]. Low ABI has also been shown to be an independent adverse prognostic factor in patients with acute ischemic stroke [37]. Therefore, the obtained results should be interpreted in such a way that ABI is such a sensitive and specific marker of subclinical CV dysfunction that a subclinical increase in hs-cTnT values in patients without AH is significantly associated with a decrease in ABI values, although it is not significantly associated with the values of other parameters of subclinical CV dysfunction.

Another interesting aspect of our results is the attempt to understand why the relationship between resting hs-cTnT and IMT is more pronounced when measured in the femoral arteries than in the carotid arteries. It is worth noting that similar conclusions have already been obtained by other researchers in various clinical contexts. Ho et al. noted that in children with type 1 diabetes, the percentage of glycated hemoglobin is a good predictor of IMT in the femoral arteries, but not in the carotid arteries [38]. According to another study, the IMT value is higher in patients with coronary artery disease compared to those without coronary artery disease, but the difference is more pronounced when IMT is measured in the femoral arteries than in the carotid arteries [39]. Further research is needed to better understand the differences in the utility of IMT measurement in different vascular beds.

Considering the mechanisms that may lead to the observed associations remains an important issue. The mechanisms leading to the release of troponin into the bloodstream from cardiomyocytes, in both physiological and pathological states, have been widely discussed. Among the proposed cellular mechanisms of troponin release, myocardial cell renewal, apoptosis, secretion of cTn degradation fragments, elevated cell wall permeability, the formation and excretion of membranous blebs, and myocyte necrosis are indicated [40,41,42,43]. More severe features of subclinical atherosclerosis, such as intima–media thickening and decreased ankle–brachial index, may also be associated with more severe subclinical coronary atherosclerosis, as atherosclerosis is a systemic process [44,45]. Even subclinical atherosclerosis is associated with decreased endothelial function [46], which leads to reduced arterial dilation in response to increased wall forces [47], which in turn can contribute to more severe subclinical cardiomyocyte damage [48]. Increased vascular stiffness contributes to left ventricular overload, which promotes the development of myocardial hypertrophy and diastolic dysfunction, which translates into increased oxygen demand and a greater predisposition to subclinical cardiomyocyte damage [49,50,51].

In this study, data were collected only on serum cardiac troponin T (cTnT) levels. Serum cardiac troponin I (cTnI) levels were not measured. Although both cTnT and cTnI are used in clinical practice, it is worth noting the key differences between these parameters. cTnI is more specific for myocardial injury than cTnT. cTnI reaches a higher peak during acute myocardial injury but normalize more quickly than cTnT. Although cTnT seems to be better suited to serve as a screening marker of CV system damage, it would be worthwhile to examine the reproducibility of the observed associations using cTnI measurements [52].

### 4.3. Strengths and Limitations of This Study

The strength of this study is the simultaneous use of different diagnostic methods relating to the status of the CV system, i.e., echocardiography, assessment of the IMT in various vascular beds, ABI, and TBI, and the measurement of PWV. To the best of our knowledge, this is the first analysis available in the scientific literature regarding the relationship between resting cTn and parameters of subclinical CV dysfunction in patients with and without AH. Another advantage of this study is the relatively homogeneous group of patients with a relatively good CV condition (the vast majority of the study participants had no diagnosed ASCVD). Furthermore, it should be noted that despite the retrospective nature of this study, there is a relatively low percentage of missing data in the collected dataset. It is also worth noting that the presented study analyzed real-world data, which allows for a better relation of the obtained results to the context of everyday clinical practice.

A limitation of this study is the relatively small group of study participants. In addition, it was a retrospective cross-sectional study, which allows only for the existence of certain correlations, without the possibility of assessing the existence of a cause/effect relationship. The study group was too small to conduct additional subgroup analyses to accurately assess the quality of hypertension control and to take into account the antihypertensive treatment used. Another significant limitation was the lack of 24-h blood pressure monitoring. Furthermore, a significant limitation is the lack of troponin I assay. Another limitation of this study is the lack of use of the latest echocardiographic techniques for assessing systolic function, such as global longitudinal strain (GLS). Finally, a further limitation is the lack of assessment of the urine albumin/creatinine ratio (UACR) (the assessment of renal function was based only on serum creatinine concentration).

### 4.4. Future Perspectives

Although our study, the results of which we presented in this publication, provides some contribution to the understanding of the relationship between resting hs-cTnT and features of subclinical CV dysfunction depending on the coexistence of AH, further research is necessary to translate the identified relationships into routine clinical practice. Prospective studies with significantly larger cohorts are needed to verify how the identified associations translate into quality of life, risk of CV events, and risk of death from CV causes. Finally, it would be necessary to verify whether incorporating resting hs-cTnT values into CV risk assessment and translating this assessment into more restrictive control of blood pressure and other modifiable CV risk factors, such as glycemia and lipid profile parameters, could improve the quality of care for patients in terms of preventing CV events, reducing the risk of CV death, and thus extending life expectancy and quality of life.

## 5. Conclusions

According to our study results, resting hs-cTnT determined without the clinical context of acute illness correlates with features of subclinical CV dysfunction, and this relationship is more pronounced in patients with AH than in those without AH. In patients with AH, a significant correlation was found between hs-cTnT and such parameters as LAVI, TRV_max_, cIMT, sfIMT, cfIMT, cSBP, cPP, PWV, and ABI. In the multivariate analysis model (adjusted for age, sex, BMI, WHtR, TC, HDL-C, LDL-C, TG, non-HDL-C, and smoking), relationships between hs-cTnT and LAVI, cSBP, cPP, ABI, and sfIMT (borderline significance) remained significant. In patients without diagnosed AH, hs-cTnT correlated significantly with sfIMT and cfIMT, whereas in multivariate analysis models, the relationship between hs-cTnT and ABI was found to be significant. Interaction analysis confirmed that AH significantly influenced the relationship between hs-cTnT and cSBP, cPP, and sfIMT.

It is worth emphasizing that although the conducted study contributes to improving our understanding of the relationship between resting hs-cTnT and features of subclinical CV dysfunction depending on the coexistence of AH, further studies are necessary to verify whether screening based on the determination of cTn could translate into a change in routine clinical practice and lead to a reduction in the risk of CV events and CV mortality. Further research is needed in this area, especially prospective studies on a larger group of patients. It would also be valuable to investigate the extent to which the troponin assay methodology may influence the conclusions obtained.

## Figures and Tables

**Table 1:** Baseline characteristics of the study population.

Variable		N (%)/Mean ± SD/Median (Q1; Q3)
Age [years]		58.35 ± 16.31
Gender		Females: 63 (62.38%) Males: 38 (37.62%)
Hypertension		65 (64.36%)
Diabetes		28 (27.72%)
Prediabetes		15 (14.85%)
Smoking	Current smoker	23 (22.77%)
	Former smoker	33 (32.67%)
	Never	42 (41.58%)
Metabolic syndrome		49 (48.5%)
Chronic kidney disease		4 (3.96%)
Atherosclerosis		67 (66.34%)
Percutaneous coronary intervention (PCI) in the past		5 (4.95%)
Coronary-aortic bypass grafting (CABG) in the past		4 (3.96%)
Stroke in the past		3 (2.97%)
Atrial fibrillation		10 (9.9%)
Body mass index (BMI) [kg/m^2^]		27.25 ± 5.05
Waist-to-hip ratio (WHR)		0.91 ± 0.098
Waist-to-height ratio (WHtR)		0.57 ± 0.094
Total cholesterol (TC) [mg/dL]		185.66 ± 41.85
Low-density lipoprotein cholesterol (LDL-C) [mg/dL]		116.17 ± 38.63
High-density lipoprotein cholesterol (HDL-C) [mg/dL]		54.71 ± 14.87
Triglycerides (TG) [mg/dL]		121.0 (77.0; 164.0)
Non-HDL-C [mg/dL]		130.95 ± 40.17
Uric acid [mg/dL]		5.13 ± 1.43
Glycated hemoglobin [%]		5.64 (5.39; 5.99)

Abbreviations: N—number; SD—standard deviation; Q1—first quartile; Q3—third quartile; PCI—percutaneous coronary intervention; CABG—coronary-aortic bypass grafting; BMI—body mass index; WHR—waist-to-hip ratio; WHtR—waist-to-height ratio; TC—total cholesterol; LDL-C—low-density lipoprotein cholesterol; HDL-C—high-density lipoprotein cholesterol; TG—triglycerides.

**Table 2:** Values of selected parameters relating to the condition of the cardiovascular system in the entire study population (without division into subgroups).

Variable	Minimal Value	Maximal Value	Mean ± SD/ Median (Q1; Q3)
hs-cTnT [pg/mL]	3.0	27.6	6.9 (4.1; 10.3)
LV EF [%]	40.0	62.0	55 (54.0; 58.0)
LAVI [mL/m^2^]	12.52	51.51	25.32 (19.95; 29.44)
LVMI [g/m^2^]	45.72	130.13	86.57 ± 16.34
E/E’	3.54	29.33	6.71 (5.9; 8.25)
TRV_max_ [m/s]	1.3	3.1	2.1 (1.8; 2.4)
cIMT [mm]	0.45	1.315	0.815 (0.65; 1.03)
cfIMT [mm]	0.38	3.95	0.85 (0.615; 1.435)
sfIMT [mm]	0.3	3.4	0.608 (0.5; 0.733)
cSBP [mmHg]	91.0	173.0	118.0 (107.0; 128.0)
cDBP [mmHg]	51.0	107.0	77.16 ± 10.21
cPP [mmHg]	16.0	91.0	39.0 (31.0; 51.0)
PWV [m/s]	4.0	23.2	7.8 (6.7; 10.2)
ABI	0.58	1.29	1.14 (1.05; 1.20)
TBI	0.33	1.17	0.8 ± 0.18

Abbreviations: SD—standard deviation; Q1—first quartile; Q3—third quartile; hs-cTnT—high-sensitivity cardiac troponin T serum concentration; LV EF—left ventricle ejection fraction; LAVI—left atrium volume index; LVMI—left ventricle mass index; E/E’—ratio of the E wave velocity value in the mitral inflow profile to the E’ wave velocity value of the mitral annulus motion in tissue Doppler echocardiography (average value of the values measured for the lateral and medial parts of the mitral annulus); TRV_max_—maximal velocity of tricuspid regurgitation; cIMT—carotid intima–media thickness; cfIMT—common femoral intima–media thickness; sfIMT—superficial femoral intima–media thickness; cSBP—central systolic blood pressure; cDBP—central diastolic blood pressure; cPP—central pulse pressure; PWV—pulse wave velocity; ABI—ankle–brachial index; TBI—toe–brachial index.

**Table 3:** Basic characteristics of patients with and without arterial hypertension (AH).

Variable	Patients with AH		Patients Without AH		*p*
	N	Mean (SD)/Median (Q1; Q3)/N (%)	N	Mean (SD)/Median (Q1; Q3)/N (%)	
Age [years]	65	67.88 (56.53; 74.51)	36	47.22 (37.01; 54.42)	<0.001 *
Percentage of females	65	53.85%	36	77.78%	0.017 ***
BMI [kg/m^2^]	64	28.86 ± 4.74	36	24.40 ± 4.31	<0.001 **
WHR	63	0.95 ± 0.09	36	0.85 ± 0.08	<0.001 **
WHtR	63	0.6 ± 0.08	36	0.5 ± 0.09	<0.001 **
TC [mg/dL]	65	180.17 ± 42.72	36	195.58 ± 38.86	0.076 **
LDL-C [mg/dL]	65	110.0 (81.6; 140.0)	36	120.0 (97.25; 141.15)	0.144 *
HDL-C [mg/dL]	65	52.83 ± 13.5	36	58.12 ± 16.72	0.087 **
TG [mg/dL]	65	126.0 (98.0; 168.0)	36	95.0 (36.0; 142.5)	0.012 *
Non-HDL-C [mg/dL]	65	127.34 ± 40.56	36	137.46 ± 39.19	0.227 **
Uric acid [mg/dL]	64	5.7 (4.85; 6.45)	36	4.2 (3.2; 4.9)	<0.001 *
Glycated hemoglobin [%]	65	5.75 (5.55; 6.09)	35	5.4 (5.24; 5.66)	<0.001 *

Abbreviations: AH—arterial hypertension; N—number; SD—standard deviation; Q1—first quartile; Q3—third quartile; BMI—body mass index; WHR—waist-to-hip ratio; WHtR—waist-to-height ratio; TC—total cholesterol; LDL-C—low-density lipoprotein cholesterol; HDL-C—high-density lipoprotein cholesterol; TG—triglycerides; *p*—*p*-value according to U Mann–Whitney test (*), Student’s *t* test (**), or χ^2^-test (***).

**Table 4:** Values of selected parameters relating to the condition of the cardiovascular system in patients with and without arterial hypertension (AH).

Variable	Patients with AH		Patients Without AH		*p*
	N	Mean (SD)/Median (Q1; Q3)/N (%)	N	Mean (SD)/Median (Q1; Q3)/N (%)	
hs-cTnT [pg/mL]	65	8.7 (6.1; 11.0)	36	3.65 (3.0; 5.6)	<0.001 *
LV EF [%]	65	55.0 (52.0; 57.0)	36	58.0 (55.0; 60.0)	<0.001 *
LAVI [ml/m^2^]	64	27.53 (22.82; 32.3)	36	23.04 (17.41; 24.93)	<0.001 *
LVMI [g/m^2^]	64	90.47 ± 16.02	36	79.62 ± 14.68	0.001 **
E/E’	65	7.07 (6.19; 8.44)	36	6.38 (5.21; 7.83)	0.026 *
TRV_max_ [m/s]	53	2.1 (1.8; 2.4)	25	1.9 (1.7; 2.3)	0.223 *
cIMT [mm]	61	0.95 (0.8; 1.065)	36	0.64 (0.583; 0.75)	<0.001 *
cfIMT [mm]	54	1.16 (0.7; 1.635)	35	0.65 (0.515; 1.015)	<0.001 *
sfIMT [mm]	53	0.65 (0.585; 0.835)	35	0.5 (0.435; 0.55)	<0.001 *
cSBP [mmHg]	65	123.0 (117.0; 132.0)	36	109.0 (100.5; 116.5)	<0.001 *
cDBP [mmHg]	65	76.98 ± 11.45	36	77.47 ± 7.6	0.819 **
cPP [mmHg]	65	46.0 (36.0; 58.0)	36	31.0 (28.0; 36.5)	<0.001 *
PWV [m/s]	62	9.35 (7.7; 11.0)	36	6.45 (5.85; 7.3)	<0.001 *
ABI	65	1.13 (1.01; 1.175)	36	1.173 (1.095; 1.218)	0.023 *
TBI	65	0.78 ± 0.19	36	0.81 ± 0.16	0.734 **

Abbreviations: AH—arterial hypertension; N—number; SD—standard deviation; Q1—first quartile; Q3—third quartile; hs-cTnT—high-sensitivity cardiac troponin T serum concentration; LV EF—left ventricle ejection fraction; LAVI—left atrium volume index; LVMI—left ventricle mass index; E/E’—ratio of the E wave velocity value in the mitral inflow profile to the E’ wave velocity value of the mitral annulus motion in tissue Doppler echocardiography (average value of the values measured for the lateral and medial parts of the mitral annulus); TRV_max_—maximal velocity of tricuspid regurgitation; cIMT—carotid intima–media thickness; cfIMT—common femoral intima–media thickness; sfIMT—superficial femoral intima–media thickness; cSBP—central systolic blood pressure; cDBP—central diastolic blood pressure; cPP—central pulse pressure; PWV—pulse wave velocity; ABI—ankle–brachial index; TBI—toe–brachial index; *p*—p-value according to U Mann–Whitney test (*) or Student’s *t* test (**).

**Table 5:** Comparison of correlation of high-sensitivity cardiac troponin T serum concentration (hs-cTnT) with selected parameters of cardiovascular dysfunction between people with and without arterial hypertension (AH) based on calculation of Spearman’s rank correlation coefficient (R).

Variable	Patients with AH			Patients Without AH		
	N	*R*	*p*	N	*R*	*p*
LV EF [%]	65	−0.22	0.079	36	−0.18	0.282
LAVI [mL/m^2^]	64	0.39	0.002	36	−0.06	0.743
LVMI [g/m^2^]	64	0.108	0.395	36	0.05	0.774
E/E’	65	0.189	0.132	36	0.26	0.121
TRV_max_ [m/s]	53	0.397	0.003	25	−0.23	0.274
cIMT [mm]	61	0.4	0.001	36	0.31	0.069
cfIMT [mm]	54	0.384	0.004	35	0.417	0.013
sfIMT [mm]	53	0.352	0.01	35	0.57	<0.001
cSBP [mmHg]	65	0.34	0.006	36	0.16	0.337
cDBP [mmHg]	65	−0.07	0.573	36	0.07	0.669
cPP [mmHg]	65	0.354	0.004	36	0.285	0.092
PWV [m/s]	62	0.63	<0.001	36	0.19	0.264
ABI	65	−0.28	0.024	36	−0.19	0.280
TBI	65	−0.07	0.580	36	−0.22	0.2

Abbreviations: AH—arterial hypertension; LV EF—left ventricle ejection fraction; LAVI—left atrium volume index; LVMI—left ventricle mass index; E/E’—ratio of the E wave velocity value in the mitral inflow profile to the E’ wave velocity value of the mitral annulus motion in tissue Doppler echocardiography (average value of the values measured for the lateral and medial parts of the mitral annulus); TRV_max_—maximal velocity of tricuspid regurgitation; cIMT—carotid intima–media thickness; cfIMT—common femoral intima–media thickness; sfIMT—superficial femoral intima–media thickness; cSBP—central systolic blood pressure; cDBP—central diastolic blood pressure; cPP—central pulse pressure; PWV—pulse wave velocity; ABI—ankle–brachial index; TBI—toe–brachial index.

**Table 6:** General linear model for multivariable analyses. Model was outcome-variable = sex + age + BMI + WHtR + TC + HDL-C + LDL-C + TG + non-HDL-C + smoking + AH + hs-cTnT + AH × hs-cTnT.

Variable	R^2^	F	*p*	*p* Interaction
LAVI [mL/m^2^]	0.32	3.67	<0.001	0.29
Log (TRV_max_)	0.21	1.57	0.13	0.086
cIMT [mm]	0.59	10.95	<0.001	0.55
cfIMT [mm]	0.45	5.75	<0.001	0.19
sfIMT [mm]	0.41	4.77	<0.001	0.052
cSBP [mmHg]	0.4	5.29	<0.001	0.011
cDBP [mmHg]	0.22	2.26	0.018	0.22
cPP [mmHg]	0.55	9.8	<0.001	0.037
PWV [m/s]	0.52	8.29	<0.001	0.1
Log (ABI)	0.32	3.76	<0.001	0.48

Abbreviations: BMI—body mass index; WHtR—waist-to-height ratio; TC—total cholesterol; HDL-C—high-density lipoprotein cholesterol; LDL-C—low-density lipoprotein cholesterol; TG—triglycerides; AH—arterial hypertension; hs-cTnT—high-sensitivity cardiac troponin T; LAVI—left atrium volume index; TRV_max_—maximal velocity of tricuspid regurgitation; cIMT—carotid intima–media thickness; cfIMT—common femoral intima–media thickness; sfIMT—superficial femoral intima–media thickness; cSBP—central systolic blood pressure; cDBP—central diastolic blood pressure; cPP—central pulse pressure; PWV—pulse wave velocity; ABI—ankle–brachial index.

**Table 7:** Generalized linear model for multivariate analysis of relationship between high-sensitivity cardiac troponin T serum concentration (hs-cTnT) and subclinical cardiovascular dysfunction in patients without arterial hypertension (AH). The model was adjusted for age, sex, BMI, WHtR, TC, LDL-C, HDL-C, TG, non-HDL-C, and smoking.

Variable	R^2^	Model (Omnibus) *p*	β	95% CI	*p* for hs-cTnT
LAVI [mL/m^2^]	0.51	0.047	−0.053	−0.42; 0.31	0.77
Log (TRV_max_)	0.62	0.14	−0.52	−1.17; 0.14	0.11
cIMT [mm]	0.60	0.0079	−0.084	−0.41; 0.25	0.605
cfIMT [mm]	0.66	0.0024	−0.031	−0.34; 0.28	0.84
sfIMT [mm]	0.57	0.018	0.0018	−0.35; 0.35	0.99
cSBP [mmHg]	0.47	0.086	−0.23	−0.61; 0.15	0.22
cDBP [mmHg]	0.57	0.013	0.20	−0.54; 0.14	0.24
cPP [mmHg]	0.46	0.097	−0.14	−0.52; 0.24	0.45
PWV [m/s]	0.36	0.34	0.20	−0.22; 0.62	0.34
Log (ABI)	0.57	0.015	−0.56	−0.91; −0.22	0.0025

Abbreviations: BMI—body mass index; WHtR—waist-to-height ratio; TC—total cholesterol; LDL-C—low-density lipoprotein cholesterol; HDL-C—high-density lipoprotein cholesterol; TG—triglycerides; LAVI—left atrium volume index; TRV_max_—maximal velocity of tricuspid regurgitation; cIMT—carotid intima–media thickness; cfIMT—common femoral intima–media thickness; sfIMT—superficial femoral intima–media thickness; cSBP—central systolic blood pressure; cDBP—central diastolic blood pressure; cPP—central pulse pressure; PWV—pulse wave velocity; ABI—ankle–brachial index.

**Table 8:** Generalized linear model for multivariate analysis of relationship between high-sensitivity cardiac troponin T serum concentration (hs-cTnT) and subclinical cardiovascular dysfunction in patients with arterial hypertension (AH). Model was adjusted for age, sex, BMI, WHtR, TC, LDL-C, HDL-C, TG, non-HDL-C, and smoking.

Variable	R^2^	Model (Omnibus) *p*	β	95% CI	*p* for hs-cTnT
LAVI [mL/m^2^]	0.27	0.13	0.40	0.053; 0.74	0.020
Log (TRV_max_)	0.23	0.48	0.23	−0.17; 0.63	0.25
cIMT [mm]	0.42	0.0047	−0.062	−0.39; 0.26	0.70
cfIMT [mm]	0.42	0.012	0.27	−0.092; 0.62	0.14
sfIMT [mm]	0.35	0.063	0.35	−0.014; 0.72	0.059
cSBP [mmHg]	0.39	0.0059	0.42	0.11; 0.74	0.009
cDBP [mmHg]	0.33	0.033	0.14	−0.19; 0.47	0.39
cPP [mmHg]	0.51	0.000094	0.33	0.048; 0.61	0.023
PWV [m/s]	0.44	0.0025	0.19	−0.14; 0.53	0.25
Log (ABI)	0.40	0.0046	−0.44	−0.75; −0.13	0.0067

Abbreviations: BMI—body mass index; WHtR—waist-to-height ratio; TC—total cholesterol; LDL-C—low-density lipoprotein cholesterol; HDL-C—high-density lipoprotein cholesterol; TG—triglycerides; LAVI—left atrium volume index; TRV_max_—maximal velocity of tricuspid regurgitation; cIMT—carotid intima–media thickness; cfIMT—common femoral intima–media thickness; sfIMT—superficial femoral intima–media thickness; cSBP—central systolic blood pressure; cDBP—central diastolic blood pressure; cPP—central pulse pressure; PWV—pulse wave velocity; ABI—ankle–brachial index.

## Data Availability

The data presented in this study are available upon request from the corresponding author.

## Abbreviations

The following abbreviations are used in this manuscript:

ABI	Ankle–brachial index
AH	Arterial hypertension
BMI	Body mass index
CABG	Coronary-aortic bypass grafting
cDBP	Central diastolic blood pressure
cfIMT	Common femoral intima–media thickness
cIMT	Carotid intima–media thickness
cPP	Central pulse pressure
cSBP	Central systolic blood pressure
cTn	Cardiac troponin
cTnT	Cardiac troponin T
cTnI	Cardiac troponin I
CV	Cardiovascular
E/E’	Ratio of the E wave velocity value in the mitral inflow profile to the E’ wave velocity value of the mitral annulus motion in tissue Doppler echocardiography (average value of the values measured for the lateral and medial parts of the mitral annulus)
HDL-C	High-density lipoprotein cholesterol serum concentration
hs-cTnT	High-sensitivity cardiac troponin T serum concentration
LAVI	Left atrium volume index
LDL-C	Low-density lipoprotein cholesterol serum concentration
LV EF	Left ventricle ejection fraction
LVMI	Left ventricle mass index
PCI	Percutaneous coronary intervention
PWV	Pulse wave velocity
Q1	First quartile
Q3	Third quartile
SD	Standard deviation
sfIMT	Superficial femoral intima–media thickness
TBI	Toe–brachial index
TC	Total cholesterol serum concentration
TG	Triglyceride serum concentration
TRV_max_	Maximal velocity of tricuspid regurgitation
WHtR	Waist-to-height ratio
WHR	Waist-to-hip ratio
